# Prophylaxis Treatment as Applied to Teeth in the Mouth

**Published:** 1905-02-15

**Authors:** D. D. Smith


### PROPHYLAXIS TREATMENT AS APPLIED TO TEETH IN THE MOUTH.

BY D. D. SMITH, D.D.S., M.D.

To understand what the prophylaxis treatment in its application to the mouth and teeth is, we must understand the long train of unstudied and disregarded pathologic conditions which have their origin in the undisturbed infection on and about the teeth in the human mouth.

Calcific deposits are constantly occurring, and not less the more immediately hurtful acidulated bacterial accumulations; and more dangerous still the inspissated mucus which cements mouth fluids, mucoid excretions, decomposing food particles, and other septic and odoriferous matter upon the teeth; this debris is all maintained in the high normal heat of the mouth—98.6 deg. F.—and furnishes ideal conditions for the proliferation of germs, the induction of decay, and the fostering of disease. The pathologic states of the mouth and teeth in the order of their seriousness and frequency, may be defined as follows: Pericemento-alveolar inflammations, dental caries and impeded alveolar development.

Resulting from the first and most important we find gum inflammations, alveolar absorption, pyorrhea, stomatitis and pericemental abscess; the latter a serious condition, resulting in the inevitable loss of the tooth. Caries, with its attendant decalcification of dentin, followed by pulp exposure, alveolar abscess, and final destruction of the *crown* of the tooth, although it has engrossed the thought of dentistry in the past and is the embodiment of its efforts in the present, is far less serious in its results. Impeded alveolar development, a common result of mouth infection of childhood, is a cause of many alveolar deformities and many crowded and irregular conditions which detract from the charm of facial expression, increase infection, incite decay and loss of the teeth.

Every human being with natural teeth is a sufferer from mouth infection, some to a greater extent than others—the civilized more than the savage.

The oral prophylaxis treatment has for its one object and aim the freeing of the oral cavity of conditions of tooth decay and the eradication of infection, to conserve health and prolong human life. In all conditions the treatment has demonstrated its sufficiency and its reasonable practicability. The benefits accruing to the mouth and teeth, and to the general health, are startling.

I maintain that tooth decay always begins on some surface of the tooth which is exposed to the fluids of the mouth. It never originates in the substance of the tooth unexposed, either crown or root. If the decay is in the crown, it begins in the enamel; if devoid of enamel, it may attack the surface of the dentin at any point. Once in the dentin, the decay proceeds along the lines of the tubules in the direction of the pulp. The process of tooth-solution is largely chemical, and is hindered or opposed by two conditions only: (*a*) the composition or consolidation of enamel and dentin, (*b*) the expression of vital energy interposed by a living pulp—a force often scarcely appreciable. No matter what the physical state of the crown of the tooth, whether hard or soft, good or bad, alive or dead, the agents and agencies which cause decay are in its environment.

The all-important matter for dentists to consider and determine is, whether oral prophylaxis, understood and properly instituted, will arrest, prevent, or retard decay of the teeth *in the mouth*. To this end there is needed not *theorizing* but clear clinical observation and close study of mouth conditions.

Ten years' experimentation has abundantly demonstrated that while the treatment will not arrest decay which is already in progress whether as new cavities or under old or imperfect fillings, it will prevent its appearance on surfaces where the prophylaxis treatment has been regularly instituted. It will also greatly retard the progress of decay in open cavities and under old fillings.

No adequate consideration has ever been given to the sources of infection inherent in the human mouth, consequent upon the presence of natural teeth. The adverse *local* consequences—decay, alveolar necrosis, pyorrhea, gum recession and final loss of the teeth—are as nothing in comparison with the evils to be revealed in the systemic disturbances and diseases which result from the continuous state of infection found in association with the teeth in the human mouth. These conditions, studied by dentists and intelligently set forth to "the mass of people," would find "a receptiveness" which would not only astonish the opponents of this "system" of preventive treatment, but would render incalculable benefits to general health, longevity, and the happiness of civilized humanity.

In general, the treatment consists of enforced, radical and frequent change of environment for all teeth and all mouth conditions, and the maintenance of perfect sanitation for the oral cavity. More in detail it is the careful and complete removal of all concretions, calcic deposits, semi-solids, bacterial plaques and inspissated secretions and excretions which gather on the surfaces of the teeth, between them, or at the gum margins; this instrumentation to be followed *in every case* by the thorough polishing of all tooth surfaces by *hand methods—power polishers should never be used*—not alone the more exposed labial and buccal surfaces of the teeth, but the lingual, palatal and proximal surfaces as well, using for this purpose orange-wood points in suitable porte polishers, charged with finely-ground pumice stone as a polishing material. Treated in this manner the teeth are placed in the most favorable condition to prevent and repel septic accumulations and deposits, and what is of equal importance, it aids the patient in all efforts to maintain sanitation and cleanliness.

A coated tongue, especially if accompanied with tonsillar inflammation, may very properly and advantageously be disinfected (mopped off) by the use of germicidal applications on bibulous paper. I have produced marked beneficial results, when finding the tongue infectiously coated through mopping it off once or twice a day with phenol sodique.

Three to six months of treatment consistently carried forward will effectually change the whole appearance, and to some extent, the whole character of a set of teeth. This change is seen in all cases, young and old, but it is specially marked in children and youth and in young adults. The dull, opaque and lifeless aspect of the teeth exhibited in ordinary conditions of nearly all mouths, quickly gives way, under the prophylaxis treatment, to a clear, pure tooth color; the teeth in a limited time become naturally translucent, and present the appearance and true characteristics of living, healthy organs. Teeth that appear as a disfigurement in the mouth, even to disgust and loathing, when subjected for a time to the prophylaxis treatment become strikingly ornate and attractive. The osseous structures, after a few months, exhibit unmistakably a marked change for the better, and in most cases they become wholly immune to decay. The vital forces within the tooth—the pulp—and those surrounding it—the pericementum and its connections—are stirred and stimulated to new life and activity,

It is most interesting and instructive to witness the awakening and revivifying of the life forces of the teeth under treatment, as exhibited in the discharge of this undue and disagreeable color, in the brightening general aspect, and in the cessation of hyper-sensitiveness and irritability.

An irritating, life-destroying infection continuously adherent to the surfaces of untreated teeth, becomes a condition of violent exhibition in some cases, and when trained to recognize it, it is distinctly manifest in all. This infection retards circulation, hinders nutrition and greatly interferes with the general health of the teeth. Manipulative treatment and the medication attending its frequent removal, stimulates and fosters the remarkable changes and improvements noticed in the character and substance of all teeth which are the subject of this treatment.

If time would permit, instances might be specifically cited and multiplied in which *no* new decay has appeared in mouths, even with complete sets of teeth, for four, five and six years; and many others in which but a modicum of decay has been found in the same length of time, and that chiefly around gold fillings.

The deciduous teeth in all cases are quickly and markedly influenced by the treatment. Of the limited number of children under seven years of age who are under the prophyaxis treatment, not one presents a new decay or a new defect n a tooth.

### AN INTERESTING CASE.

One remarkably-interesting and instructive case is that of a boy, a strongly-marked nervous temperament, brought to me when but three and a half years of age, in delicate health. At this time there were five large cavities—three approximal—in his teeth, and two places exhibiting such predisposition to decay that I prognosed cavities in them within three months. Through exercise of patience and perseverance the cavities were excavated and filled with amalgam. (Perfect operations were impossible.) Immediately following the fil'ing, for an entire year, his teeth were carefully treated every two weeks, barring one month during the summer vacation. Since that time—nearly five years—I have seen him on an average of once a month; meanwhile his teeth have received rather more than the ordinary care of chilhood, at home. The boy is now in his ninth year, with this record: Excessive nervousness arrested; general health fully established; teeth, as to decay, in perfect condition (predicted cavities did not appear); one new pin-head cavity in distal sulcus of left superior temporary molar (probably started before first five fillings were made); one re-decay around largest and most difficult of the five original fillings. Twelve of the temporary teeth have loosened and come out, every one in nature's own way, through complete root absorption. First permanent molars, inferior and superior permanent incisors erupted, cuspids presenting. The first molars—erupted at five and a half—have unusually large crowns with strongly-marked, pointed cusps and correspondingly deep sulci and linear markings. Formation, shape, and time of eruption of these teeth, presaged early and rapid decay; nevertheless, they are at this writing all in perfect state of preservation, and evidently improving in character. Neither infection nor chemical agents are permitted to fasten upon any of their surfaces, and the life forces are evidently building into them a more compact and decay-resisting structural consolidation.

The benefits resulting from the prophylaxis treatment as exhibited in this case are equally marked in every case where deciduous teeth have been subjected to the treatment.

As a further observation of the benefits of this treatment, I desire to put on record a development of marked significance, namely, a decided modification noticed in the sensitiveness of the dentin of teeth, especially in children, following the institution of a regular and consistent treatment. Diminishing of acute sensitiveness seems to follow as a general result, and not to be some special temporary condition. The extreme sensitiveness in the dentin of young teeth has been so modified and lessened, apparently by this treatment, as that ordinary operations for filling have been performed without the intense suffering usually attending such cases. Children and young people who are under this treatment submit to ordinary operations without compulsion or a special complaint. Operations, that under the old regime would cause much suffering, if not deemed unbearable, have been greatly mitigated in intensity.

Hyper-sensitiveness of dentin is a result of pericemental irritation far more than of pulp irritation. The external and most important life of the tooth—the pericemental life—is markedly influenced by the irritative infection found always at the necks of untreated teeth. Removal of this infection is the removal of the cause of much of the undue sensitiveness of dental tissue.

The benefits which accrue directly to the teeth are not more manifest nor more pronounced than such as immediately appear in the pericementum and gums. The presence of irritative infectious matter on the surfaces of untreated teeth, sufficient to affect the pericementum and surrounding gum tissue, is found in connection with all untreated teeth. It appears coeval with their eruption, and continues, in ordinary conditions, as long as the teeth are retained. It is bounded only by the extent of the dentate surface exposed in the mouth; as a result, the pericemental tissue and gums in ordinary mouth conditions are perpetually in a state of undue sensitiveness, and not less, a condition of undue, often extreme, vascularity. Under the prophylaxis treatment, through which all irritants are removed, these tissues quickly lose their extreme and unnatural sensitiveness, and recover the normal condition of low-grade sensibility. The circulatory vessels also lose the unnatural distension and tumefaction consequent upon irritation, recover tonicity, and contract to normal dimensions. Three to six months of treatment will remove all marked sensibility and tendency to bleed, and operations for filling at cervical margins, especially on the upper jaw, can be safely and easily performed without the use of the rubber dam. It is the irritative, toxic matter at the necks of the teeth that occasions the sensitive states and highly-vascular conditions of the gums and pericemental tissue. A mouth with teeth freed from this infection and kept in an aseptic condition will have gums tense and hard, without vascularity and without undue sensibility.

We have, then, to commend the prophylaxis treatment: first, for its prevention of decay; second, for its stimulation to external and internal life and health of the teeth; third for its influence in decreasing sensation in dentin and gum tissue; fourth, for its correction of the undue vascularity in gum tissue, and fifth, for its positive removal of all infection from the mouth and ultimately from the breadth.

It may be well to enter somewhat into detail respecting the care and co-operation expected, *even required*, of patients under the prophylaxis treatment. In the beginning, for four to six months they are required to present regularly, on appointment, once a month for treatment, which is carefully applied, using only hand methods as heretofore described.

The tooth-brush, which is the main reliance of the patient, is as best an inefficient instrument for cleansing teeth. It is, however, the best our civilization has produced; hence, until something better is devised, necessity compels the use of it.

It may be said, without hesitation or fear of contradiction, there is no part of the human body which is so imperfectly and ignorantly cared for as this most important cavity containing the teeth. Attending to the teeth by the patient is a matter which has neither been studied nor taught either by the professionalist or the laity. The child is *taught* to wash the hands, to bathe the body—but the human mouth, the very vestibule of life, is left almost wholly without intelligent care.

The results are everywhere seen in repulsive exhibits of decayed, infection-coated teeth, offensive breaths, a display of discolored fillings, and that dental monstrosity, so common—the gold crown.

The general public, compelled to rely on the tooth-brush, is yet without proper equipment for cleansing the teeth. The whole operation soon becomes distasteful, irksome, and generally the merest farce. Patients present with mouths reeking with infection, teeth loaded with deposits that are antiquated, and affirm "that they are very careful about brushing their teeth."

All this should be changed. It can only be done by a process of education, and that through instruction given by dentistry. Every dentist should first inform himself, and then fully instruct individual patients in the details of the care of the human mouth. The people should be taught that cleaning the teeth is not merely the possession of a tooth-brush. The best shaped brush in the world will not clean the teeth in a human mouth unless it is intelligently used.

In shape it should be plain and perfectly straight, the bristles neither coarse nor fine, set in rows of different lengths, in substance all of the best materials. There should be no concavity to fit the arch when the brush is at rest; no fancy curves; no tufts of bristles at the end. It should be of size, in length and width, to form a mass of bristles to cover the teeth and give stability and substance when in use.

In using the brush it should be passed over labial, buccal, lingual and palatal—or the whole outer and inner surfaces of the teeth, with a vigorous *horizontal* movement, the hand adapting the brush, as far as possible, to all tooth surfaces. With care and a straight brush all parts of the exposed crowns may thus be reached and cleansed as well as a brush can do this office work. Attempting to cleanse the teeth with the vertical movement, using a concave brush, as some teach, on the convex surface of the dental arches, while of little avail, will do no harm, either to the teeth or gums. Such use of the brush in a measure relieves the interstices of food remains, and thus assists in a work that can be much better done with a tooth-pick.

It has been urged that the vertical movement of the brush acts as a kind of massage, tending to brush the gums onto the teeth, while the horizontal movement brushes them away from the teeth. This is the merest *theory*, a statement wholly without justification in actual conditions. The vertical movement of the brush is not more a massage of the gums than is the horizontal movement. There is no process of brushing which approximates a massage treatment of the gums, and it would avail nothing if there could be. My patients are always instructed, in connection with the prophylaxis treatment, to use the brush with a vigorous horizontal movement, even when the gums and the alveolar process have necrosed until the festoons have become everted and to continue the brushing until the teeth are not only brushed but *cleansed*. I have never yet seen a case in which reformation of tissue has not followed the treatment, and in all cases not connected with pyorrhea, restoration of the festoons has been complete and perfect.

The tooth-brush implies a dentifrice, and a dentifrice is demanded for cleaning the teeth.

Something of the powder or soap nature is demanded always, with the brush. Patients should be taught to avoid all pastes or liquid dentifrices, for they are not only inefficient, but frequently harmful. A good soap, as a pure Castile, may be used *ad libitum*, and always with benefit to the mouth.

The impression very generally prevails that the *time* for brushing the teeth is after meals. This is a fallacy for dentistry to uproot. To receive the greatest benefit from the use of the tooth-brush, the mouth should be thoroughly cleansed always just *before* meals. Infection gathers upon the teeth in the interim between meals, when the salivary glands are at rest and the teeth not in use; it is during periods of rest that the mucous secretion and mucoid excretions from mucous surfaces are lodged upon the teeth in greatest quantity. Cleansing the mouth and teeth from this viscid debris, *before* meals, prevents the washing of these toxic tooth accretions into the gastro-intestinal tract with the food. Who is not taught to wash the *hands* before meals? Of how much greater importance is the cleansing of the human mouth! If the accustomed method with the brush is not practicable, it may be always practicable to thoroughly rinse the mouth with water and to wipe the main exposed surfaces of the teeth with the corner of a towel or napkin. This method of cleaning the teeth is not enough in vogue. After meals, the teeth may be freed of food remains with some form of tooth-pick, preferably the "quill," which is all that is required. Careful cleansing, devoting not seconds but minutes to the operation, should always precede retiring for the night and immediately succeed rising in the morning.

Alveolar pyorrhea is not properly classified as a "disease." Its very definition—flow of pus—implies that it should be taken from the category of diseases and placed among the inflammations. Properly defined, alveolar pyorrhea is an inflammation within the confines of the pericemental membrane and contiguous tissues; an inflammation due wholly to the irritation resulting from stagnant septic matter adherent to the surfaces, necks and roots of natural teeth.

A typical case of pyorrhea: A married man in middle life—thirty-eight years—with hard, strong teeth, crowns large and irregular, roots not long and probably conical; marked cervical constriction, and pericemental tissue scanty.

This gentleman is a smoker, but not given to excesses or vices; had brushed his teeth latterly with so-called "prophylactic" brush after approved methods, vertical movement.

The broad-bladed scaler of the Smith set was passed back to the lingual surface of the second lower molar, where it was loaded with years of accumulations of calcic toxins, recent exudations of globules of pus, and gatherings of every variety of mouth debris. This conglomerate mass of revolting matter was held up to view, and the patient asked if he would like to handled it, to smell it, to taste it, to have it returned to the mouth. To all of these suggestions there was prompt declination. Then it was clearly explained that matter of similar nature is found on all untreated teeth, and that teeth are not on the outside of the body, *but inside the mouth;* that such conditions exist, not one hour in the day, but twenty-four hours of every day; not one day in the year, but three hundred and sixty-five days of every year; and further, this infectious matter on the teeth *in the mouth,* is in a temperature constantly maintained at 98.6 deg. F., than which nothing could possibly be more favorable for germ culture, for imparting odors to the breath, nor for filling the lungs with infectious emanations.

From the mouth and teeth, septic matter is being conveyed directly into the gastro-intestinal tract, thence to to the circulation, whence it may be deposited in any organ or tissue, and become the nucleus of serious chronic, systemic maladies. To emphasize the continual presence of this deleterious matter, the patient was told he could, at any hour of the day, draw floss silk between the teeth, or pass the finger over their surfaces, and discover most repellent odors; or he could recall the unpleasant taste in the mouth on rising in the morning, the fetid breath, especially at night, due not to stomach conditions, but to the stagnant, odoriferous matter on the teeth. Becoming dry, through inactivity of the salivary glands, the teeth send out into the breath of the sleeper poisonous emanations to do continuously a work of infection, unnoticed and unsuspected.

I would that it might be recognized by all, that sequences of the universal infection on the teeth in the oral cavity are, in our present civilization, affecting the health of humanity to a greater extent than any other one physical condition. I could wish nothing grander or better for our profession than that it should see and embrace the magnificent opportunity unfolded in oral prophylaxis to move forward and occupy this new field of untold benefits.

The secrets of tooth-decay in and of themselves are of minor consequence in comparison with the systemic infections due directly to states and conditions of the teeth in the mouth. We venture to predict that recognition will ere long be made of the fact that tuberculosis—that decimator of homes—and possibly other grave chronic disorders, can find no soil for development in a system kept from the contaminations of mouth-infection through a vigorous prophylaxis treatment from childhood.

We have abundantly proven that diabetes and many gastro-intestinal troubles are directly traceable to the mouth infection of alveolar pyorrhea; also that many pharyngeal and tonsilar inflammations, and many skin troubles have their origin in infection of the mouth, due to septic states of neglected teeth.

We know also that mental depression and hypochondria and many of the perplexing nervous conditions of women result from the same cause. Dentistry alone has the power to relieve humanity from the plague of mouth infection When shall it command its own rightful place among the specialties of medicine?—*Dominion Journal.*

